# Health literacy in higher education students: findings from a Portuguese study

**DOI:** 10.1093/eurpub/ckac130.140

**Published:** 2022-10-25

**Authors:** AR Pedro, R Rosário, I Monteiro, M Cerqueira, S Roque, V Assunção, D Brandão, A Escoval, PL Ferreira

**Affiliations:** 1 Public Health Research Centre, National School of Public Health of NOVA University of Lisbon, Lisbon, Portugal; 2 Comprehensive Health Research Centre, Lisbon, Portugal; 3 School of Nursing, University of Minho, Braga, Portugal; 4 Health Sciences Research Unit: Nursing, Nursing School of Coimbra, Coimbra, Portugal; 5 University of Aveiro, Aveiro, Portugal; 6 Escola Superior de Saúde de Portalegre, Instituto Politécnico de Portalegre, Portalegre, Portugal; 7 Faculty of Dental Medicine, University of Lisbon, Lisbon, Portugal; 8 Faculty of Economics, University of Coimbra, Coimbra, Portugal; 9 Centre for Health Studies and Research of the University of Coimbra, Coimbra, Portugal; 10 Rede Académica de Literacia em Saúde, Lisbon, Portugal

## Abstract

**Background:**

Health literacy (HL) concerns the knowledge and competences of people to meet the complex demands of health in modern society. It is essential for health promotion, disease prevention and healthcare. Young adults can perform a very important role in taking a more active role in managing and protect their health, so this study aimed to identify the HL levels in the population of higher education students, according to the European Health Literacy Survey (HLS-EU-PT) and to evaluate its association with social and academic determinants.

**Methods:**

A quantitative, observational, and cross-sectional study was carried out based on an online survey disseminated in Portuguese universities. Data were analysed using binary logistic regression, adjusted for age, income, parents’ education, gender, and chronic disease report.

**Results:**

In total, 4801 students were surveyed, 76% female. Of those, 44% revealed a problematic or inadequate level of HL. Those students with higher income levels (OR (95% CI), OR = 4.5 (3.4; 5.9) and whose parents had higher education levels (OR = 1.3(1.1; 1.5) had higher odds of achieving sufficient or excellent levels of HL, even after adjusting for confounders. In what concerns academic determinants, data revealed that HL tends to be sufficient or excellent among those students from health-related courses (OR = 2.0 (1.6; 2.5). In the subgroup of students from non-health-related courses, it was found that HL levels do not differ in 1st year and last year's students. However, in students from health-related courses, data revealed that a last yeaŕs students had higher odds of having sufficient or excellent HL levels compared to a 1st-year student (OR = 1.7 (1.4; 2.2).

**Conclusions:**

This study reveals low HL levels and addresses that socioeconomic and familiar context are determinants of HL in higher education students. Future intervention studies are needed, focused on these determinants so that adequate levels of HL are achieved in higher education students.

**Key messages:**

- Almost half of the higher education students had inadequate or problematic levels of health literacy.
- Both socioeconomic, familiar, and academic contexts are significantly associated with health literacy levels among higher education students.

